# Exploring the Association between Aggression and Suicidal Thoughts and Behaviors in an Urban Pediatric Primary Care Setting

**DOI:** 10.3390/psychiatryint7030122

**Published:** 2026-06-02

**Authors:** Andrea S. Young, Emily T. O’Gorman, Eleanor G. Wu, Laura Prichett, Robert Yolken, Emily Severance, Juleisa Badio, Destini Carmichael, Tina Kumra

**Affiliations:** 1 Department of Psychiatry and Behavioral Sciences, Johns Hopkins School of Medicine; 2 Department of Mental Health, Johns Hopkins Bloomberg School of Public Health; 3 Department of Pediatrics, Johns Hopkins School of Medicine

**Keywords:** Pediatric primary care, suicide risk, youth depression, aggressive behavior

## Abstract

Rates of suicide among children and adolescents have increased significantly in the past two decades, especially among minoritized youth. Identification of modifiable factors associated with suicidality in diverse samples is important for informing targeted prevention and intervention efforts. Toward this end, this study utilized a multi-informant approach to examine the association between aggression and suicide risk in a urban pediatric sample. Children and adolescents (*N*=136; 69% Black or African American) between the ages of 6 and 17 (*Mage* = 11.4 ± 3.0) were recruited while attending a well-child visit at a Baltimore City pediatric primary care clinic. Pediatric participants and their caregivers completed measures of aggressive behavior and depressive problems. Suicide risk was derived from parent-, youth-, and clinician-report of pediatric participants experiencing suicidal thoughts and behaviors. After controlling for demographic variables, results of stepwise logistic regressions revealed that parent- and youth-reported aggressive behavior were significantly associated with suicide risk (OR= 1.18, p =.005 and OR = 1.23, p = .006, respectively). When depressive problems were added to the model, depressive problems were significantly associated with suicide risk (parent-report OR= 1.34, p =.015 and youth-report OR = 1.28, p = .025), but aggressive behavior was no longer significantly associated. Findings from this study suggest that aggression could be an important indicator of suicide risk but not above and beyond the influence of depressive symptoms. In this sample, aggressive behavior may be at least partially explained by depressive symptoms and a manifestation of internal distress.

## 1. Introduction

Rates of suicide and suicide attempts among children and adolescents have increased significantly in the past two decades, with the steepest rises among racial and ethnic groups that historically have decreased access to mental health care [1–6]. Further, community-level research within historically marginalized populations is funded at a notably lower rate than research in other topic areas [7] underscoring the need for research focused on mental health care access and risk factors among diverse populations and to expand culturally sensitive prevention and intervention efforts [8]. Racial disparities in service access are generally less pronounced in primary care than in specialty mental health care settings [9]; therefore, primary care may represent a critical point of contact for early suicide risk identification and intervention for children and adolescents who may have decreased access to mental health care. Studying risk factors associated with suicidality within pediatric primary care samples can inform scalable prevention strategies that reach patients who may not otherwise engage in specialty services.

Several studies have explored relationships between suicidality and one such factor: trait or reactive aggression. Aggression [10] is a leading cause of pediatric referrals for urgent psychiatric consultation [11]. While it is not specific to any diagnosis, it is often characterized as impulsive/reactive (a response to a stimulus, which can include “outbursts” or “tantrums”) or proactive (premeditated) [12]. Children and adolescents with elevated levels of impulsive/reactive aggression may be more likely to have ADHD, mood disorders, and substance use [13]. Irritability, which typically refers to a mood (e.g., anger, frustration), is highly associated with and often precedes aggression [14, 15] and is also associated with suicidality [16, 17]. Aggression (or expressed anger), and suicidality could be linked through a shared diagnostic driver (e.g., depression) [16], shared genetic risk [18], or shared biological mechanism (e.g., behavioral approach vs. inhibition) [16, 19]. Retrospective studies of youth who died by suicide have suggested that higher levels of aggression are associated with increased suicide risk [20, 21]. In community samples, aggression is associated with increased risk of suicidal ideation and attempts among adolescents [22]. A recent meta-analysis found a moderate correlation between suicide and aggression in adolescents across studies [23]. Research examining the association between suicidality and aggression has primarily been among predominantly white samples. One longitudinal study within a predominantly Black community sample found teacher-reported aggressive behavior to be significantly associated with student-reported suicide attempts in females [24]. Of note, however, this study did not examine other potential precursors to suicide attempts (e.g., suicidal ideation) and utilized teacher-report, which was largely based on school observations. Examining suicidality through other means, including self- and caregiver-report, offer additional opportunities to understand suicidality in community sample children and adolescents from groups with historically limited access to mental health care. Further, exploring these questions in pediatric primary care samples is especially valuable because of the implications for scalable screening and brief intervention.

Studies suggest that the relationship between aggression and suicide is moderated by several factors, including severity of externalizing [25, 26] and internalizing [27, 28] symptoms, trauma [29] and general stress [27]. More specifically, reactive aggression has been associated with suicide risk among children and adolescents with higher (but not lower) levels of externalizing symptoms [25]. Similarly, among adolescents with higher levels of internalizing symptoms or general stress, aggressive behavior has been shown to be a positive predictor of suicidal ideation [27]. With respect to trauma, among preadolescents and adolescents, genetic risk for impulsive aggression interacted with higher levels of traumatic experiences to predict suicidal ideation over and above anxiety and depression [29].

The severity and growing nature of the public health issue of suicide among minoritized children and adolescents makes identification of individual differences associated with suicidality among diverse samples particularly important, as these could be key targets for prevention and intervention programs in pediatric primary care. Toward this end, the current study aimed to add to the extant literature by utilizing multiple methods to examine the relationship between aggression and suicide risk in an urban pediatric primary care clinic serving with a patient population predominantly identifying as Black. As past research indicates that reports from different informants (e.g., parents, youth, clinicians) capture meaningful differences in aspects of a child’s functioning [30, 31], pediatric psychopathology was assessed via both parent- and youth-report, and suicidal thoughts and behaviors was derived from parent-, youth-, and clinician-report. Our primary questions were: is aggression associated with suicidality among patients at an urban pediatric primary care clinic; and, if so, does that association remain when adjusting for depressive symptoms?

## 2. Materials and Method

### 2.1. Participants

Participants included 136 patients from the Pediatric COVID Antibody Study, originally designed to investigate the long-term effects of COVID-19 infection on mental health in a pediatric primary care population being seen in a community clinic in a large mid-Atlantic city. Patients ages 6–17 years (Mage = 11.4, SD = 3.0) attending a well-child visit that included a blood draw between 2/1/2022 and 11/21/2022 were invited to participate in the study. Patients’ caregivers also consented to allow the study team access to their medical records. Patients and caregivers provided assent and consent, respectively. The majority of the study sample (69%) reported identifying as Black or African American, followed by 22% white, 7% multiracial or biracial, <1% Asian or Pacific Islander, and 2% who declined to answer. Almost half (47%) of participants resided in neighborhoods with an Area Deprivation Index (ADI) in the top 20th percentile in the state, and 28% of participants were at or below the state poverty line.

This study was reviewed and approved by the Institutional Review Board. All legal guardians provided written consent; children/adolescents provided written assent. Children/adolescents and caregivers received gift cards in exchange for their participation.

### 2.2. Measures

#### 2.2.1. Aggressive Behavior and Depressive Problems.

Caregivers completed the Child Behavior Checklist (CBCL), which is a valid and reliable measure of child psychiatric symptoms and functioning across multiple domains [32]. Youth who were ages 11 to 17 at the time of their initial visit and were willing and able to complete forms (n = 75) completed the parallel self-report form, the Youth Self Report (YSR) [33]. Of primary interest in the present study were the Aggressive Behavior and Depressive Problems scales on both the CBCL and the YSR. While both scales were examined continuously in the primary analyses, for descriptive purposes, we also report the proportions of youth about the cutoff for clinical significance which is a T-score of 70 or above.

#### 2.2.2. Suicide Risk

A multi-method suicide risk variable was derived from two caregiver-report indicators, three youth-report indicators, and two clinician-report indicators. The seven indicators contributing were as follows: 1) caregiver endorsement of the CBCL suicidal thoughts item (talking about suicide); 2) caregiver endorsement of the CBCL self-injurious behaviors/suicide attempts item; 3) youth endorsement of suicidal thoughts on the YSR; 4) youth-report of self-injurious behaviors/suicide attempts on the YSR; 5) youth endorsement of item 9 on the Patient Health Questionnaire (PHQ-9) [34], which addresses suicidal thoughts and is administered as part of routine care in pediatric primary care; 6) clinician-report of the patient having a history of suicide attempt or suicidal ideation, per their medical records; and 7) record of an emergency department visit with behavioral health as the primary concern, per the patient’s medical record. The dichotomous suicide risk variable was coded as 1 when one or more of these indicator variables were present; absence of any indicators was coded as 0.

#### 2.2.3. Demographics

Race, ethnicity, age and gender were self-reported and extracted from the electronic medical record. Area Deprivation Index (ADI) was also extracted from the electronic medical record based on participants’ home addresses. The ADI is a validated, neighborhood-level index of communities’ socioeconomic disadvantage incorporating information about employment, income, housing, and education [35, 36]. As previously noted, nearly 70% of the sample identified as Black or African American. Due to this and our interest in suicide risk among Black or African American youth, race was dichotomized to reflect identifying as Black/African American or another race (white, Asian, Indigenous/American Indian or Alaska Native, more than one race). No one in the sample identified as Hispanic or Latino; as such, ethnicity was not included in the analyses.

### 2.3. Data Analyses

Descriptive statistics and bivariate correlations among key variables were computed in SPSS. For the purpose of characterizing the sample in a meaningful manner, T-scores for CBCL and YSR scales were used when computing descriptive statistics. Subsequent regression analyses utilized raw scores, with participants’ age and gender included in the regression equations to control for these variables.

Multivariable logistic regression analyses were utilized to examine the association between aggressive behavior and suicide risk. Separate models were run for parent-reported and youth-reported depressive problems and aggressive behavior. In both models, age and gender were entered at Step 1 to examine the relationship between these variables and suicide risk and to adjust for the influence of these variables in the model. At Step 2, race (coded as Black or a different race) and ADI were entered to examine and adjust for their effects after controlling for age and sex. At Step 3, CBCL or YSR Aggressive Behavior was entered to test for the main effect of this variable on suicide risk. In the last step, CBCL or YSR Depressive Problems was entered to examine its main effect after controlling for other variables in the model and to determine whether any relationship between Aggressive Behavior and suicide risk would exist with depression included in the model. All analyses were completed in SPSS with p <.05 as the cutoff for statistical significance.

Of 136 participants, only 2 (1.5%) were missing data on variables of interest (one was missing CBCL scores and one was missing an ADI). Given the low level of missingness, those participants were excluded from analyses including those variables.

## 3. Results

Descriptive statistics and correlations among key variables are reported in Table 1. Overall, the sample endorsed average levels of aggressive behavior and depressive problems. Parent and youth reported problems were correlated with each other. At least one suicide risk indicator was endorsed by approximately 10% of the sample (n = 14; 2 had a history of suicide attempt in their medical record; 6 endorsed suicidality on the PHQ-9; 7 reported deliberate self-harm and 8 reported thoughts about suicide on the YSR; 7 of children/adolescents’ caregivers reported deliberate self-harm and 7 reported thoughts about suicide on the CBCL). Depressive problems and aggressive behavior were associated with suicide risk across both parent-and youth-reports (ps<.001). Of note, suicide risk was not significantly associated with race, gender, or area deprivation index. Older patients were more likely to have evidence of suicide risk. Neither parent-nor youth-reported depressive problems or aggressive behavior were significantly associated with race, age, gender, or area deprivation index.

Results of logistic regression models using parent- and youth-report are presented in Table 2. In the parent-report model, older age was significantly associated with suicide risk (OR=1.33, p=.009). Demographic covariates were not significantly associated with suicide risk in the youth-report model. Using both parent-reported and youth-reported symptoms, aggressive behavior was significantly associated with suicide risk at Step 3 (OR= 1.18, p =.005 and OR = 1.23, p = .006, respectively). However, when depression was entered at Step 4, depressive problems were significantly associated with suicide risk (parent-report OR= 1.34, p =.015 and youth-report OR = 1.28, p = .025, respectively), and aggressive behavior was no longer significantly associated.

To better contextualize these findings, we examined mean T-scores and proportions of patients reaching the clinical cut off for parent- and youth-reported depressive problems and aggressive behavior (Table 3). Mean T-scores were in the average range for patients without evidence of suicide risk. Mean T-scores for patients with suicide risk were in the CBCL/YSR “borderline” range of 65–69 for parent-reported depressive problems. Among patients with suicide risk, 21–42% had clinically elevated (T-score ≥ 70) aggressive behavior and 43–50% had clinically elevated depressive problems, while proportions of patients with clinically elevated aggressive behavior or depressive problems without suicide risk fell between 3 and 11%.

## 4. Discussion

This analysis sought to fill a critical gap in the literature by examining potential modifiable risk factors associated with suicide related outcomes in a predominantly Black pediatric sample in a primary care setting, a population and context in which this topic has historically been understudied. This study further extended previous research by examining symptoms using both parent and youth self-report and multiple methods to assess suicide risk behaviors. It was hypothesized that, after controlling for demographic predictors (i.e., age, sex, race, ADI), aggression would be positively associated with suicidal thoughts and behaviors.

Our hypothesis was partially supported. Aggressive behavior was associated with suicide risk in demographic-adjusted models; however, this association was no longer statistically significant after depressive problems were added, suggesting that aggression may operate as a clinical marker of suicide risk that is not independent of co-occurring depressive symptomatology. This pattern aligns with prior longitudinal research indicating that conduct and aggression symptoms may not uniquely predict suicidality when depressive symptoms are modeled concurrently [37]. These findings have clinically relevant implications for pediatric primary care, a key access point for identifying mental health problems and suicide risk. Unfortunately, depression can be difficult to detect in pediatric primary care [38]. This challenge may be especially pronounced for Black children and adolescents, who may be more likely to experience depression that presents as irritability, anger, or behavioral dysregulation rather than sadness and withdrawal [39], which may lead providers to interpret their symptoms as “behavior problems” rather than signs of a depressive episode. Indeed, Black children and adolescents are more often diagnosed with disruptive behavior disorders and less often with mood disorders [40, 41], suggesting that behavior manifestations of internal distress may be more readily pathologized than the internal distress itself in this population. In addition to annual depression screening as recommended by the American Academy of Pediatrics for mitigating suicide risk [42], our results suggest that aggression may be a useful flag for prompting (1) a more targeted assessment of depressive symptoms that includes irritability and related affective presentations and (2) direct suicide risk screening, given guidance emphasizing that depression screening alone may miss some youth at suicide risk [43].

Importantly, this is a pediatric primary care sample (not a sample selected for mental health risk), with mean depressive problems and aggressive behavior scores falling in the normal range. This both makes the sample ideal for informing how risk might be identified in primary care settings and contributed to the fairly low frequency of suicide risk. We may have been underpowered to detect a main effect of aggression on suicide risk outcomes while adjusting for the effect of depressive symptoms. Further, the low base rate of suicide risk indicators in our sample combined with the number of covariates and predictors in our models may have reduced the precision of logistic regression estimates, including the possibility of inflated odds ratios. Findings should therefore be interpreted as preliminary and replicated in larger primary care samples with more youth endorsing suicide risk.

The present findings should be considered in light of a few other limitations. This study was conducted in Baltimore City, Maryland, a medium-sized urban area; our findings may not generalize to more rural or suburban areas. In addition, several modifiable risk factors of suicidality were not measured in this study, including but not limited to: trauma exposure, substance use, sleep/circadian factors (Arrona-Palacios et al., 2021) and peer relationships. These variables may confound or mediate the relationship between aggressive and depressive symptoms with suicidality and future studies should pursue a better understanding of how context and symptomatology interact to shape suicide risk. Further, our sample spanned a wide developmental age range. Although age was included as a covariate and was not significantly associated with depressive problems or aggressive behavior, older age was associated with greater likelihood of suicide risk, and independently predicted suicide risk in the parent report model. This finding is consistent with increases in suicidal thoughts and behaviors observed in adolescence, and it is plausible that the relationship between aggression, depression, and suicide risk may vary across developmental periods. Given our sample size, we were unable to examine these developmental differences directly, and this is a topic that warrants future research for informing suicide screening efforts in pediatric primary care. Finally, the analyses described in this manuscript are cross-sectional; thus, we cannot determine temporality between depression, aggression, and suicide risk. Future longitudinal studies are needed to determine whether aggression and depressive symptoms precede, co-occur with, or follow the emergence of suicide risk among urban pediatric primary care samples.

This study also has several important strengths. First, a multi-method approach to assessing pediatric psychopathology and suicide risk was utilized. While there is a relatively low rate for suicide risk in this sample, it is high compared to studies in a larger pediatric primary care sample operationalizing suicide risk using a single indicator (e.g., suicide-related diagnosis in the medical record; [44]. This suggests that having additional indicators (parent-report on the CBCL, child-report on YSR and PHQ-9, clinician-report from the medical record) provided a broader scope of suicide risk related behaviors than reviewing the electronic medical record alone. This multi-indicator, multi-informant approach may have decreased the likelihood of missing a patient at risk for suicide [45]. Second, 70% of the sample for this study were Black or African American children and adolescents, a group historically underrepresented in clinical research and in suicide risk research specifically. Of note, we found no significant differences in suicide risk, depressive problems or aggressive behavior by race suggesting that depressive problems and aggressive behavior impact children and adolescents similarly across race in this setting. Thus, this study contributes to the existing literature by demonstrating the effects of depression and aggression on suicide in a predominantly Black or African American pediatric sample. Further, the sample consisted of patients presenting to a pediatric primary care clinic, as opposed to children and adolescents presenting for inpatient or outpatient mental health services; thus, it highlights the importance of screening mental health problems among pediatric patients in this setting and treating and/or referring these patients to specialty mental health care.

## 5. Conclusions

Findings from this study highlight aggressive behavior, in the presence of elevated depression, as a modifiable risk factor associated with suicidality among a predominantly Black pediatric primary care sample. Of note, in this sample, aggressive behavior may be at least partially explained by depressive symptoms and a manifestation of internal distress.

As there are different types of aggression (impulsive vs. proactive), future research might examine whether there is a particular type or subset of aggressive behavior that is most strongly associated with suicide risk behaviors. Future research should also examine longitudinal associations between aggression, depression, and suicide risk behaviors.

## Figures and Tables

**Table 1.** Descriptive statistics and bivariate correlations among key variables

	M (SD)	1	2	3	4	5	6	7	8
1. Age	11.4 (3.0)								
2. Gender	74 (54.4%)^a^	.12							
3. Race	94 (59.1%)^b^	**−.23**	.10						
4. ADI Score (State; N=135)	7.61 (2.5)	.09	.04	**−.63**					
5. CBCL Depressive Problems (N=134)	55.7 (8.0)	*.21*	−.003	.02	−.003				
6. CBCL Aggressive Behavior (N=134)	53.6 (6.3)	.11	.04	.001	.03	**.62**			
7. YSR Depressive Problems (N=75)	56.1 (7.4)	*.23*	.02	.16	−.15	**.67**	**.35**		
8. YSR Aggressive Behavior (N=75)	54.5 (6.7)	.17	−.06	.17	−.14	**.42**	**.43**	**.45**	
9. Suicide Risk	14 (10.3%)^c^	**.25**	.12	−.02	.05	**.44**	**.32**	**.55**	**.42**

*Note. N*=136 unless otherwise noted;

a *n* (%) female;

b *n* (%) Black;

c *n* (%) suicide flag.

ADI= Area Deprivation Index; CBCL = Child Behavior Checklist; YSR= Youth Self-Report.

Bold font indicates correlation is significant at *p* < .01; Italicized font indicates correlation is significant at *p* < .05; CBCL and YSR *M*s and *SD*s are for T-scores.

**Table 2.** Aggressive behavior and depressive problems in relation to suicide risk

Step Parameter	Parent-Report Model (N=134)					Youth Self-Report Model (ages 11+ only; N=75)				
	B	OR	OR 95% CI		*p*	B	OR	OR 95% CI		*p*
			Lower	Upper				Lower	Upper	
1										
Age	0.28	1.33	1.07	1.64	**.009**	0.23	1.26	0.90	1.77	.171
Gender	0.58	1.78	0.51	6.25	.369	0.28	1.32	0.34	5.18	.694
2										
Race	0.85	2.33	0.39	14.00	.354	0.71	2.03	0.22	18.91	.535
ADI	0.15	1.17	0.82	1.66	.392	0.08	1.09	0.73	1.62	.677
3										
Aggressive Behavior	0.17	1.18	1.05	1.33	**.005**	0.21	1.23	1.06	1.43	**.006**
4										
Aggressive Behavior	0.03	1.03	0.88	1.21	.688	0.11	1.12	0.94	1.32	.207
Depressive Problems	0.29	1.34	1.06	1.69	**.015**	0.25	1.28	1.03	1.59	**.025**

*Note*. ADI = Area Deprivation Index; OR = Odds ratio.

**Table 3.** Mean T-Scores for aggressive behavior and depressive problems and proportion of patients with clinically elevated scores in relation to suicide risk

	Suicide Risk (parent-report n=14; youth-report n=12)		No Evidence of Suicide Risk (parent-report n=134; youth-report n=75)	
	T-score, M (SD)	Clinically Elevated, n (%)	T-score, M (SD)	Clinically Elevated, n (%)
**Parent-report**				
Aggressive behavior	59.5 (10.6)	3 (21.4%)	52.9 (5.2)	7 (5.9%)
Depressive problems	65.9 (13.0)	6 (42.9%)	54.5 (6.3)	12 (10.2%)
**Youth-report**				
Aggressive behavior	60.8 (10.9)	5 (41.7%)	53.3 (4.8)	2 (3.2%)
Depressive problems	63.7 (11.7)	6 (50.0%)	54.6 (5.3)	6 (9.5%)

## Data Availability

These data are part of an ongoing study and include protected health information; therefore, they are not available to persons outside of the study team.

## Abbreviations

The following abbreviations are used in this manuscript:

ADI: Area Deprivation Index
CBCL: Child Behavior Checklist
CI: Confidence interval
OR: Odds ratio
PHQ-9: Patient Health Questionnaire - 9
SD: Standard deviation
YSR: Youth Self-Report

## References

[1] Bridge JA, , Suicide trends among elementary school-aged children in the United States from 1993 to 2012. JAMA Pediatrics, 2015. 169(7): p. 673–677. 25984947 10.1001/jamapediatrics.2015.0465

[2] Wadsworth T, Kubrin CE, and Herting JR, Investigating the Rise (and Fall) of Young Black Male Suicide in the United States, 1982–2001. Journal of African American Studies, 2014. 18(1): p. 72–91.

[3] Bridge JA, , Youth Suicide During the First Year of the COVID-19 Pandemic. Pediatrics, 2023. 151(3). 10.1542/peds.2022-058375 PMC10227859 36789551

[4] Ryan SC, , Disparities in suicide risk trajectories among youth during the COVID −19 pandemic. Journal of research on adolescence., 2026. 36(1). 10.1111/jora.70132 PMC12835459 41588576

[5] Akhtar M, , Racial disparities in suicide-related mortality in the US: Examining trends before, during, and after the COVID-19 pandemic using the CDC WONDER database. Psychiatry research, 2025. 356: p. 116885. 41389656 10.1016/j.psychres.2025.116885

[6] Young AS, , Adequacy of Children’s Psychopharmacology Services: Variations by Race and Clinical Characteristics. Psychiatric Services, 2023. 10.1176/appi.ps.20220375 PMC10983772 37287230

[7] Hoppe TA, , Topic choice contributes to the lower rate of NIH awards to African-American/black scientists. Science Advances, 2019. 5(10). 10.1126/sciadv.aaw7238 PMC6785250 31633016

[8] The Congressional Black Caucus Emergency TaskForce on Black Youth Suicide and Mental Health, Ring the Alarm: The Crisis of Black Youth Suicide in America. 2020.

[9] Smith LBOB,C; Wei K; Kenney G; Waidman T, Well-Child Visits in Medicaid in 2019 and 2020. 2024, Urban Institute: Washington, DC.

[10] Young AS, , Developing and Validating a Definition of Impulsive/Reactive Aggression in Youth. J Clin Child Adolesc Psychol, 2020. 49(6): p. 787–803. 31343896 10.1080/15374416.2019.1622121 PMC6980978

[11] Pikard J, Roberts N, and Groll D, Pediatric Referrals for Urgent Psychiatric Consultation: Clinical Characteristics, Diagnoses and Outcome of 4 to 12 Year Old Children. J Can Acad Child Adolesc Psychiatry, 2018. 27(4): p. 245–251. 30487940 PMC6254263

[12] Stepanova E, , Finding a needed diagnostic home for children with impulsive aggression. Clinical Child and Family Psychology Review, 2023: p. 1–13. 10.1007/s10567-022-00422-3 36609931

[13] Youngstrom EA, , Developing Empirical Latent Profiles of Impulsive Aggression and Mood in Youths across Three Outpatient Samples. J Clin Child Adolesc Psychol, 2023. 52(2): p. 196–211. 34125637 10.1080/15374416.2021.1929251 PMC9173587

[14] Perhamus GR, Ostrov JM, and Murray-Close D, Aggression and Irritability in Middle Childhood: Between- and Within-Person Associations. J Clin Child Adolesc Psychol, 2024. 53(2): p. 184–198. 37976108 10.1080/15374416.2023.2272941 PMC11043001

[15] Quarmley M, Vafiadis A, and Jarcho JM, Irritability and rejection-elicited aggression in adolescents and young adults. Journal of Child Psychology and Psychiatry, 2023. 64(9): p. 1346–1358. 37036378 10.1111/jcpp.13804

[16] Daniel SS, , Trait anger, anger expression, and suicide attempts among adolescents and young adults: a prospective study. J Clin Child Adolesc Psychol, 2009. 38(5): p. 661–71. 20183651 10.1080/15374410903103494 PMC2854503

[17] Benton TD, , Dysregulation and Suicide in Children and Adolescents. Child and Adolescent Psychiatric Clinics of North America, 2021. 30(2): p. 389–399. 33743946 10.1016/j.chc.2020.10.008

[18] Hill SY, Jones BL, and Haas GL, Suicidal ideation and aggression in childhood, genetic variation and young adult depression. J Affect Disord, 2020. 276: p. 954–962. 32745832 10.1016/j.jad.2020.07.049 PMC7484359

[19] Martin CP, , Examining Behavioral Approach and Inhibition to Further Characterize Youth With Impulsive Aggression. JAACAP Open, 2023. 1(4): p. 263–273. 39553447 10.1016/j.jaacop.2023.08.001 PMC11562435

[20] McGirr A, , Impulsive-aggressive behaviours and completed suicide across the life cycle: a predisposition for younger age of suicide. Psychological Medicine, 2008. 38(3): p. 407–417. 17803833 10.1017/S0033291707001419

[21] Renaud J, , Current psychiatric morbidity, aggression/impulsivity, and personality dimensions in child and adolescent suicide: A case-control study. Journal of Affective Disorders, 2008. 105(1–3): p. 221–228. 17568682 10.1016/j.jad.2007.05.013

[22] Moore FR, Allott C, and O'Connor R, Impulsivity, aggression, and impulsive aggression in suicidality. Personality and Individual Differences, 2023. 202: p. 111971.

[23] Detullio D, Kennedy TD, and Millen DH, Adolescent aggression and suicidality: A meta-analysis. Aggression and Violent Behavior, 2022. 64: p. 1–15.

[24] Musci RJ, , Suicide attempt endophenotypes: Latent profiles of child and adolescent aggression and impulsivity differentially predict suicide attempt in females. Preventative Medicine Reports, 2022. 28: p. 101829. 10.1016/j.pmedr.2022.101829 PMC9126944 35620051

[25] Abel MR, , Reactive aggression and suicidal behaviors in children receiving outpatient psychological services: The moderating role of hyperactivity and inattention. Child Psychiatry and Human Development, 2020. 51(1): p. 2–12. 31222508 10.1007/s10578-019-00905-5

[26] Levy T, , Suicidality risk in children and adolescents with externalizing disorders: Symptoms profiles at high risk and the moderating role of dysregulated family relationships. European Child & Adolescent Psychiatry, 2023. 10.1007/s00787-023-02190-z 37043094

[27] Buitron V, , Aggressive behaviors and suicide ideation in inpatient adolescents: The moderating roles of internalizing symptoms and stress. Suicide and Life-Threatening Behavior, 2018. 48(5): p. 580–588. 28833393 10.1111/sltb.12375 PMC6445376

[28] Kerr DCR, , Sequelae of aggression in acutely suicidal adolescents. Journal of Abnormal Child Psychology, 2007. 35(5): p. 817–830. 17534711 10.1007/s10802-007-9132-5

[29] Koyama E, , Predicting risk of suicidal ideation in youth using a multigene panel for impulsive aggression. Psychiatry Research, 2020. 285: p. 112726. 31870620 10.1016/j.psychres.2019.112726

[30] Carlson GA and Youngstrom EA, Two opinions about one child--what's the clinician to do? J Child Adolesc Psychopharmacol, 2011. 21(5): p. 385–7. 22040183 10.1089/cap.2011.2159

[31] De Los Reyes A, Introduction to the Special Section: More Than Measurement Error: Discovering Meaning Behind Informant Discrepancies in Clinical Assessments of Children and Adolescents. Journal of Clinical Child and Adolescent Psychology, 2011. 40(1): p. 1–9. 21229439 10.1080/15374416.2011.533405

[32] Ivanova MY, , Testing the 8-syndrome structure of the child behavior checklist in 30 societies. J Clin Child Adolesc Psychol, 2007. 36(3): p. 405–17. 17658984 10.1080/15374410701444363

[33] Achenbach TM and Rescorla LA, Manual for the ASEBA School-Age Forms and Profiles. 2001, Burlington, VT: University of Vermont, Research Center for Children, Youth, and Families.

[34] Kroenke K, Spitzer RL, and Williams JBW, The PHQ-9. Journal of General Internal Medicine, 2001. 16(9): p. 606–613. 11556941 10.1046/j.1525-1497.2001.016009606.x PMC1495268

[35] Kind AJH and Buckingham WR, Making Neighborhood-Disadvantage Metrics Accessible - The Neighborhood Atlas. N Engl J Med, 2018. 378(26): p. 2456–2458. 29949490 10.1056/NEJMp1802313 PMC6051533

[36] University of Wisconsin School of Medicine and Public Health. Area Deprivation Index. 2022 2022]; Available from: https://www.neighborhoodatlas.medicine.wisc.edu/.

[37] Vander Stoep A, , Risk for Suicidal Ideation and Suicide Attempts Associated with Co-occurring Depression and Conduct Problems in Early Adolescence. Suicide and Life-Threatening Behavior, 2011. 41(3): p. 316–329. 21463356 10.1111/j.1943-278X.2011.00031.x PMC3683657

[38] Kramer T and Garralda ME, Psychiatric disorders in adolescents in primary care. British Journal of Psychiatry, 1998. 173(6): p. 508–513. 10.1192/bjp.173.6.508 9926080

[39] Anderson ER and Mayes LC, Race/ethnicity and internalizing disorders in youth: A review. Clinical psychology review., 2010. 30(3): p. 338–348. 20071063 10.1016/j.cpr.2009.12.008

[40] Fadus MC, , Unconscious Bias and the Diagnosis of Disruptive Behavior Disorders and ADHD in African American and Hispanic Youth. Academic Psychiatry, 2020. 44(1): p. 95–102. 31713075 10.1007/s40596-019-01127-6 PMC7018590

[41] Liang J, Matheson BE, and Douglas JM, Mental Health Diagnostic Considerations in Racial/Ethnic Minority Youth. Journal of Child and Family Studies, 2016. 25(6): p. 1926–1940. 27346929 10.1007/s10826-015-0351-z PMC4916917

[42] Zuckerbrot RA, , Guidelines for Adolescent Depression in Primary Care (GLAD-PC): Part I. Practice Preparation, Identification, Assessment, and Initial Management. Pediatrics, 2018. 141(3). 10.1542/peds.2017-4081 29483200

[43] Mournet AM, , Limitations of Screening for Depression as a Proxy for Suicide Risk in Adult Medical Inpatients. Journal of the Academy of Consultation-Liaison Psychiatry, 2021. 62(4): p. 413–420. 34219655 10.1016/j.jaclp.2021.02.002 PMC8258235

[44] Prichett LM, , Recent anti-infective exposure as a risk factor for first episode of suicidal thoughts and/or behaviors in pediatric patients. Brain, Behavior, & Immunity - Health, 2024. 36: p. 100738. 10.1016/j.bbih.2024.100738 PMC10906143 38435723

[45] Spears AP, , Future directions in understanding and interpreting discrepant reports of suicidal thoughts and behaviors among youth. Journal of Clinical Child & Adolescent Psychology, 2023. 52(1). 10.1080/15374416.2022.2145567 PMC9898197 36473063

